# Metabolic syndrome in infertile women with polycystic ovarian syndrome

**DOI:** 10.1590/2359-3997000000135

**Published:** 2016-02-11

**Authors:** Tahereh Madani, Roya Hosseini, Fariba Ramezanali, Gholamreza Khalili, Nadia Jahangiri, Jila Ahmadi, Fatemeh Rastegar, Zahra Zolfaghari

**Affiliations:** 1 Department of Endocrinology and Female Infertility Reproductive Biomedicine Research Center Royan Institute for Reproductive Biomedicine Tehran Iran Department of Endocrinology and Female Infertility, Reproductive Biomedicine Research Center, Royan Institute for Reproductive Biomedicine, ACECR, Tehran, Iran; 2 Department of Epidemiology and Reproductive Health Reproductive Biomedicine Research Center Royan Institute for Reproductive Biomedicine Tehran Iran Department of Epidemiology and Reproductive Health, Reproductive Biomedicine Research Center, Royan Institute for Reproductive Biomedicine, ACECR, Tehran, Iran

**Keywords:** Polycystic ovary syndrome, metabolic syndrome, prevalence, ATPIII criteria

## Abstract

**Objective:**

: The aim of the present study was to determine the prevalence of metabolic syndrome (MS) in infertile Iranian women with polycystic ovary syndrome (PCOS) using the ATPIII criteria.

**Subjects and methods:**

: In this cross-sectional study, 624 women with PCOS were enrolled at a tertiary referral center in Tehran, Iran, between April, 2012 and March, 2013. Diagnosis of MS was according to ATPIII criteria. Also, we divided PCOS patients into following two main groups: (i) with MS (n = 123) and (ii) without MS (n = 501), and then compared variables between two groups.

**Results:**

: The mean age, body mass index (BMI) and waist circumference were 28.6 ± 4.3 years, 26.7 ± 3.7 kg/m^2^ and 85.2 ± 8.7 cm, respectively. The prevalence of MS was 19.7%. Our findings showed that age, BMI, waist circumference and all metabolic parameters were higher in PCOS women with MS than related values in those without MS. The most and least prevalent forms of MS were low level of high density lipoprotein-cholesterol (HDL-C) and hypertension, respectively.

**Conclusion:**

: It seems the prevalence of metabolic syndrome in our country isn’t as high as western countries. The prevalence rate of MS increased with age and BMI. One of the major cardiovascular risk factors, low level of HDL-C, is the most prevalent metabolic abnormality in our participants.

## INTRODUCTION

Polycystic ovary syndrome (PCOS) is one of the most common reproductive endocrinological disorders in women, affecting about 15% of women in general population according to Rotterdam criteria (1). Insulin resistance (IR) plays an important role in pathophysiology of PCOS (2,3). Evidence has shown that IR and compensatory hyperinsulinemia also play central roles in the evolution of metabolic syndrome (MS) (4-8). MS is a group of risk factors that identify individuals at increased risk for type 2 diabetes mellitus and atherosclerosis (9,10). These risk factors include central obesity, hypertriglyceridemia, low levels of high-density lipoprotein (HDL) cholesterol, elevated blood pressure and fasting plasma glucose levels (10). Many of the metabolic abnormalities of PCOS patients overlap with components of MS. The prevalence rates of MS in PCOS women vary among different countries and ethnicities as follows: 43-46% in America (11,12), 37.9% in India (13), 35.3% in Thailand (14), 28.4% in Brazil (15), 16.8% in China (16), 14.5% in Korea (17), 11.6% in Turkey (18) and 8.2% in Southern Italy (19). These differences in prevalence rates of MS in PCOS patients in different countries may be depended to several factors, like age, BMI, and race of patients as well as different approaches to define MS and PCOS.

To consider the various reports about the prevalence of MS among PCOS patients in different countries and the lack of evidence describing the prevalence of MS in PCOS patients in Iran, we sought to report these. The aim of the present study was to determine the prevalence of MS and its components in Iranian infertile women with PCOS using the ATPIII criteria.

## SUBJECTS AND METHODS

This cross-sectional study was conducted between April, 2012 and March, 2013, while it was approved by the Institutional Review Board and the Ethical Committee of Royan Institute Research Center according to the Helsinki Declaration. Informed consent was also signed by all participants.

### Patients

Women with PCOS attending the tertiary referral Infertility Clinic of Royan Institute, Tehran, Iran, were enrolled in this study. Diagnosis of PCOS was based on Rotterdam criteria (20). Women who were aged > 40 and who used contraceptive drugs within 3 months prior the study were excluded from the study. Based on appropriate clinical and/or laboratory tests, other causes of hyperandrogenism, such as 21-hydroxylase deficiency, Cushing’s syndrome, androgen secreting tumors, hypothyroidism, and hyperprolactinemia were also excluded from the study. So, we divided PCOS patients into following two main groups: (i) with MS (n = 123) and (ii) without MS (n = 501).

### Procedure

National Cholesterol Education Program Adult Treatment Panel (NCEP ATP III) criteria was used for the diagnosis of MS; therefore, we considered the presence of three or more of the following abnormalities to confirm a diagnosis: waist circumference ≥ 88 cm, fasting glucose ≥ 100 mg/dL, fasting serum triglycerides ≥ 150 mg/dL, serum HDL-C < 50 mg/dL, and blood pressure ≥ 130/85 mmHg (21).

For each participant weight and height were measured in light clothing without shoes. Hip and waist circumference (WC) and waist-to-hip ratio were measured as standard method. Body mass index (BMI) was calculated as body weight in kilograms was divided by the square of height in meters. BMI in this study was categorized into three groups as follows: (i) non-obese (n = 215) when BMI < 25 kg/m^2^, (ii) overweight (n = 295) when BMI 25-29.9 kg/m^2^ and (iii) obese (n = 114) when BMI ≥ 30 kg/m^2^. Age was also stratified into four subgroups: (i) < 25 years (n = 116), (ii) 25 – 29.9 years (n = 255), (iii) 30-34.9 years (n = 181) and (iv) 35-39.9 years (n = 72).

Blood pressure was measured on the right arm of women in a sitting position after 15 minutes rest using a manual mercury sphygmomanometer. Hirsutism was defined by the presence of excessive body hair in an androgen-depended pattern, using the Ferriman and Gallwey score > 8 (22). Oligomenorrhea was defined as the presence of three or more cycles of > 35 days in the previous 6 months, and amenorrhea was referred to the absence of vaginal bleeding for 3 months. Hypermenorrhea was defined as vaginal bleeding occurring at an interval of less than 21 days. Vaginal ultrasonography was performed by expert gynecologists on third day of menstrual cycle for each patient.

Blood samples were drawn after a 12-hour overnight fasting on second or third day of their spontaneous or progesterone induced menstrual cycles. Subsequently, luteinizing hormone (LH), follicle-stimulating hormone (FSH), free testosterone, dehydroepiandrosterone-sulfate (DHEA-S), 17OH progesterone, triglycerides, total cholesterol, low density lipoprotein (LDL) cholesterol, high density lipoprotein (HDL) cholesterol, fasting blood glucose and insulin, and 2-hour blood glucose (after eating 75 gram oral glucose) were carried out at the laboratory department of the Royan Institute. Non HDL cholesterol was calculated by subtracting HDL cholesterol form total cholesterol. The states of glucose tolerance were classified into four groups according to the World Health Organization (WHO) and American Diabetes Association (ADA) (23) as follows: (i) impaired fasting glucose (IFG), (n = 54) when fasting plasma glucose was between ≥ 100 mg/dL and < 126 mg/dL; (ii) impaired glucose tolerance (IGT), (n = 39) when after 120 minutes and taking 75 g anhydrous glucose, plasma glucose was between ≥ 140 mg/dL and < 200 mg/dL; as well as (iii) diabetes, (n = 11) when either fasting plasma glucose was ≥ 126 mg/dL, and/or after120 minutes and glucose load, plasma glucose was ≥ 200 mg/dL.

The homeostatic model assessment-estimated insulin resistance (HOMA-IR) is a simple method to measure IR which was calculated as fasting glucose (mg/dL) × fasting insulin (μU/mL)/405 (24).

Sample size was calculated for estimation of the prevalence of MS in PCOS women attending our infertility clinic. Data were analyzed by a software package used for statistical analysis (SPSS) version 20 (SPSS, Inc., Chicago, IL, USA). Descriptive statistics, mean ± standard deviation (SD) and frequency (%) were used to describe the characteristics of participants. The Student’s t-test was used to compare the continuous variables between groups. For comparing categorical variables, χ^2^ test was used. A p-value of < 0.05 was considered statistically significant.

## RESULTS

A total of 624 women were enrolled to study. The mean age and BMI were 28.6 ± 4.3 years and 26.7 ± 3.7 kg/m^2^, respectively. Obesity was seen in 114 (18.3%) women with PCOS, while 295 (47.3%) were overweight. According to the results of glucose metabolism, 80.1% had normal glucose metabolism, 8.7% had IFG, 6.3% had IGT, 3.2% had combined IFG and IGT, and 1.8% had diabetes mellitus.

The prevalence rates of menstrual irregularities in our patients were: oligomenorrhea (64.7%), amenorrhea (25.3%), eumenorrhea (8.4%), polymenorrhea (0.8%) and mixed pattern (0.8%). In addition, hirsutism score > 8, acne and male pattern balding were seen in 30.1%, 22.1% and 8.3% of patients, respectively. The overall prevalence of MS was 19.7 %. Also, 37.7% and 28.2% of patients showed one and two criteria for MS, respectively. Clinical and biochemical characteristics of participants are summarized in table 1, while table 2 demonstrates the prevalence of the metabolic syndrome according to different age and body mass index groups. The prevalence rates of different components of MS are shown in table 3. Our results indicated that among PCOS patients with MS, the most prevalent forms of MS components were low level of HDL cholesterol (92.8%) followed by increased WC (82.9%), whereas the least prevalent form was high blood pressure (8.1%) (Table 3).

**Table 1:** Anthropometric, hormonal, metabolic and sonographic characteristics of PCOS women with and without MS

Variables	PCOS with MS (n = 123)	PCOS without MS (n = 501)	p-Value*
Patients	19.7%	80.3%	
Age (year)	30.1 ± 4.3	28.3 ± 4.3	< 0.001
BMI (kg/m^2^)	29.8 ± 3.4	25.9 ± 3.4	< 0.001
Waist (cm)	93.2 ± 7	83.3 ± 7.9	< 0.001
WHR	0.85 ± 0.05	0.81 ± 0.05	< 0.001
Systolic pressure (mmHg)	114 ± 12.5	108.9 ± 10.5	< 0.001
Diastolic pressure (mmHg)	72.2 ± 10.6	69.1 ± 8.9	0.003
Oligomenorrhea	59.3%	66.1%	0.32
Amenorrhea	32.5%	23.6%	0.12
Hirsutism	25.0%	23.6%	0.79
**Hormonal profile**			
LH (mIU/mL)	7.5 ± 4.1	8.4 ± 5.3	0.03
FSH (mIU/mL)	6 ± 3.4	6 ± 2.4	0.86
LH/FSH	1.5 ± 1.2	1.5 ± 1.1	0.51
Free testosterone (pg/mL)	1.6 ± 1.3	1.7 ± 2.2	0.50
DHEA-S (μg/dL)	134 ± 376	139 ± 275	0.99
17 OH progesterone (ng/mL)	1.2 ± 1.1	1.4 ± 1.6	0.23
**Metabolic parameters**			
FG (mg/dL)	98.4 ± 19.2	86.7 ± 8.7	< 0.0001
Fasting insulin (mIU/mL)	13.3 ± 9.6	11.6 ± 11.7	0.17
FG/fasting insulin	12.6 ± 17.1	14.5 ± 40	0.61
HOMA-IR	3.3 ± 2.6	2.5 + 2.6	0.001
Total cholesterol (mg/dL)	192.5 ± 34.5	177.5 ± 34.3	< 0.0001
LDL-C (mg/dL)	116.4 ± 30.9	107.2 ± 29.3	0.002
HDL-C (mg/dL)	38.8 ± 9.4	45.9 ± 11.7	< 0.0001
Non-HDL-C (mg/dL)	154.6 ± 36.2	132 ± 34	< 0.0001
TG (mg/dL)	189 ± 97.4	113 ± 52.3	< 0.0001
**Sonographic parameters**			
Right ovary volume (cm^3^)	8.6 ± 3.6	8.3 ± 4	0.64
Left ovary volume (cm^3^)	9 ± 5.1	8.3 ± 4.3	0.55
Right ovary antral follicle number	17 ± 6.4	16.5 ± 6.4	0.59
Left ovary antral follicle number	17 ± 6.5	16.4 ± 5.8	0.55

PCOS: polycystic ovary syndrome; MS: metabolic syndrome; BMI: body mass index; WHR: waist-to-hip ratio; LH: luteinizing hormone; FSH: follicle stimulating hormone; DHEA-S: dehydroepiandrosterone sulfate; FG: fasting glucose; TG: triglycerides; LDL-C: low density lipoprotein cholesterol; HDL-C: high density lipoprotein cholesterol; HOMA-IR: Homeostasis Model Assessment–Insulin Resistance.

Data are presented as mean ± standard deviation or number (%).

* Comparison is performed between PCOS women with and without MS by Student t-test and Chi-square test.

**Table 2:** Prevalence of the metabolic syndrome according to different age and body mass index groups

Variable	MS/Total	Frequency (95% CI)	p-Value
Age (years)			
< 25	11/116	9.5% (4.2%-14.8%)	< 0.001
25-29.9	45/255	17.6% (13.0%-22.3%)	
30-34.9	42/181	23.2% (17.1%-29.4%)	
35-39.9	25/72	34.7% (23.7%-45.7%)	
BMI (kg/m^2^)			
< 25	7/215	3.3% (0.9%-5.6%)	< 0.001
25-29.9	64/295	21.7% (17.0%-26.4%)	
≥ 30	52/114	45.6% (36.5%-54.8%)	

MS: metabolic syndrome; BMI: body mass index.

**Table 3:** Prevalence of metabolic syndrome components* in 624 patients with PCOS

MS components	Prevalence %(n)			

	MS (n = 123)	Without MS (n = 501)		Total (n = 624)
HDL-C < 50 mg/dL	92.8 (114)		66.3 (332)	71.5 (446)
WC ≥ 88	82.9 (102)		22.8 (114)	34.6 (216)
TG ≥ 150 mg/dL	70.7 (87)		15 (75)	26 (162)
FG ≥ 100 mg/dL	43.9 (54)		5.6 (28)	13.1 (82)
BP ≥ 130/85 mmHg	8.1 (10)		0.8 (4)	2.2 (14)

PCOS: polycystic ovary syndrome; MS: metabolic syndrome; HDL-C: high density lipoprotein cholesterol; WC: waist circumference; TG: triglycerides; FG: fasting glucose; BP: blood pressure.

* According to NCEP ATPIII criteria.

## DISCUSSION

This study showed the prevalence of MS in Iranian infertile PCOS patients was 19.7% according to ATPIII criteria and Rotterdam criteria. Also, our results show that this prevalence increased with age and BMI. This prevalence is lower than related values in many American and Asian reports. For example, in several studies in US, the prevalence of MS was 33.4–46% according to NCEP-ATP III criteria (12,25). In India and Brazil, this prevalence rates were 37.9% and 28.4% (13,17), respectively. In contrary, in several European countries, the prevalence rates of MS in PCOS women are lower than our results, like 11.6% in Turkey (18), 8.2% in southern Italy according to ATPIII criteria (19) and 1.6% in the Czech Republic (26). It seems one of the important causes for discrepancy in the prevalence of MS in women with PCOS is different criteria for diagnosis of PCOS and MS. For example, Bhattacharya showed in Indian PCOS women, MS was found in 47.5% and 37.9% cases according to IDF criteria and ATP III criteria, respectively (13). Also, based on WHO criteria and ATP-III criteria, Carmina and cols. found the prevalence rates of MS were 16% and 8.2%, respectively, in Italian PCOS women (19). In addition to different criteria for diagnosis of PCOS and MS, the characteristics of the population studied, such as race, age, BMI, different dietary habits and lifestyle in different countries, had important roles for different prevalence rate of MS in PCOS women.

Another important risk factor for MS in PCOS patients and also in general populations is advance age (27-29). In our study, the mean age of subjects was 26.8 ± 4.3 years. Vural and cols. found in Turkish PCOS women – the country with similar (geographical environment and eating habits to our country – the prevalence rate of MS was lower than our study. This difference may be due to lower average age of participants in their study (21.4 ± 1.8 years) (18).

The prevalence rate of MS in our study was higher in upper age groups [9.5% (< 25 years) *vs*. 34.5% (35-40 years)]. According to results of Third National Health and Nutrition Examination Survey (NHANES) for US population, the prevalence of the MS increased with advanced age, reaching peak levels in the seventh decade for women (29). It seems increasing in prevalence of MS with advanced age is related to increased prevalence of overweight and obesity. Soares and cols. (15) reported in Brazilian women with PCOS, the prevalence of MS increased with advancing age. The cause of age related insulin resistance, however, remains unknown, Boden and cols. (30) found that at least part of the insulin resistance in aging may be due to age-related changes in body composition rather than age itself.

Obesity has a key role in evolution of MS. Our study showed in upper BMI groups, the prevalence of MS were higher [3.2 % (BMI < 25) *vs*. 46 % (BMI ≥ 30)]. The association between the prevalence of the MS and BMI was shown in normal population (29) and PCOS patients (15,25). Ehrmann and cols. showed PCOS women in the highest quartile of BMI had nearly a 14-fold increased chance of having the MS compared with women in the lowest quartile of BMI (25).

Insulin resistance and compensatory hyperinsulinemia are key pathogenetic factors in MS, but insulin levels per se are not applied for diagnosis of the MS. We found that fasting insulin levels in PCOS patients with MS were significantly higher than PCOS patients without MS. In agreement with this finding, Ehrmann and cols. showed a significant increasing trend in the proportion of women with the MS as related to the fasting insulin concentration; the prevalence of the MS from lowest to highest quartile of fasting insulin was 12.1, 25.3, 38.5, and 58.2%, respectively. This trend remained significant even after adjusting for BMI (25). Belong to Ehrmann and cols.’s study chance for having MS in the highest quartile of fasting insulin was 5-fold greater (95% CI = 2.1–11.8) than lowest quartile after adjustment for the effect of body weight (25). Also, our finding showed that one of the important IR index, HOMA-IR, was significantly higher in PCOS with MS group. In agreement with these findings, several studies show a statistically significant increase in fasting insulin level and in HOMA-IR in PCOS patients with and without MS (25,31,32).

Our results show the most prevalent factors of MS component in PCOS patients were low level of HDL cholesterol (71.5%) followed by increased WC (34.6%). However, the most prevalent forms of MS component in PCOS patients were different in previous studies. In agreement with our results, Soares and cols. (15) showed the most prevalent forms of MS components was HDL-C level < 50 mg/dL in 69.6% followed by WC ≥ 88 cm in 57.9%.

Marcondes and cols.’s study showed the best predictors of MS were a WC > 88 cm, HDL-C < 50 mg/dL and triglycerides ≥ 150 mg/dL (31). In Espinós-Gómez and cols.’s study, WC, low HDL-C and high triglyceride concentrations had a valid association for selecting PCOS patients as a good candidate for routine metabolic screening (32).

In conclusion, it seems the prevalence of MS in our country isn’t as high as western countries. The prevalence increases significantly with age and BMI. The most prevalent form in metabolic abnormality is low HDL-C.

## References

[1] . Fauser BC, Tarlatzis BC, Rebar RW, Legro RS, Balen AH, Lobo R, et al. Consensus on women’s health aspects of polycystic ovary syndrome (PCOS): the Amsterdam ESHRE/ASRM-Sponsored 3rd PCOS Consensus Workshop Group. Fertil Steril. 2012;97(1):28-38.e25. 10.1016/j.fertnstert.2011.09.024 22153789

[2] . Dunaif A. Insulin resistance and the polycystic ovary syndrome: mechanism and implications for pathogenesis. Endocr Rev. 1997;18(6):774-800. 10.1210/edrv.18.6.0318 9408743

[3] . Diamanti-Kandarakis E, Dunaif A. Insulin resistance and the polycystic ovary syndrome revisited: an update on mechanisms and implications. Endocr Rev. 2012;33(6):981-1030. 10.1210/er.2011-1034 PMC5393155 23065822

[4] . Ford ES. The metabolic syndrome and mortality from cardiovascular disease and all-causes: findings from the National Health and Nutrition Examination Survey II Mortality Study. Atherosclerosis. 2004;173(2):309-14. 10.1016/j.atherosclerosis.2003.12.022 15064107

[5] . Haffner SM, Valdez RA, Hazuda HP, Mitchell BD, Morales PA, Stern MP. Prospective analysis of the insulin-resistance syndrome (syndrome X). Diabetes. 1992;41(6):715-22. 10.2337/diab.41.6.715 1587398

[6] . Isomaa B, Almgren P, Tuomi T, Forsén B, Lahti K, Nissén M, et al. Cardiovascular morbidity and mortality associated with the metabolic syndrome. Diabetes Care. 2001;24(4):683-9. 10.2337/diacare.24.4.683 11315831

[7] . Lakka HM, Laaksonen DE, Lakka TA, Niskanen LK, Kumpusalo E, Tuomilehto J, et al. The metabolic syndrome and total and cardiovascular disease mortality in middle-aged men. JAMA. 2002; 288(21):2709-16. 10.1001/jama.288.21.2709 12460094

[8] . Trevisan M, Liu J, Bahsas FB, Menotti A. Syndrome X and mortality: a population-based study. Risk Factor and Life Expectancy Research Group. Am J Epidemiol. 1998;148(10):958-66. 10.1093/oxfordjournals.aje.a009572 9829867

[9] . Eckel RH, Grundy SM, Zimmet PZ. The metabolic syndrome. Lancet. 2005;365:1415-28. 10.1016/S0140-6736(05)66378-7 15836891

[10] . Grundy SM, Cleeman JI, Daniels SR, Donato KA, Eckel RH, Franklin BA, et al.; American Heart Association; National Heart, Lung, and Blood Institute. Diagnosis and management of the metabolic syndrome: an American Heart Association/National Heart, Lung, and Blood Institute Scientific Statement. Circulation. 2005;112(17):2735-52. 10.1161/CIRCULATIONAHA.105.169404 16157765

[11] . Glueck CJ, Papanna R, Wang P, Goldenberg N, Sieve-Smith L. Incidence and treatment of metabolic syndrome in newly referred women with confirmed polycystic ovarian syndrome. Metabolism. 2003;52(7):908-15. 10.1016/s0026-0495(03)00104-5 12870169

[12] . Apridonidze T, Essah PA, Iuorno MJ, Nestler JE. Prevalence and characteristics of the metabolic syndrome in women with polycystic ovary syndrome. J Clin Endocrinol Metab. 2005;90(4):1929-35. 10.1210/jc.2004-1045 15623819

[13] . Bhattacharya SM. Prevalence of metabolic syndrome in women with polycystic ovary syndrome, using two proposed definitions. Gynecol Endocrinol. 2010;26(7):516-20. 10.3109/09513590903367010 20540665

[14] . Weerakiet S, Bunnag P, Phakdeekitcharoen B, Wansumrith S, Chanprasertyothin S, Jultanmas R, et al. Prevalence of the metabolic syndrome in Asian women with polycystic ovary syndrome: using the International Diabetes Federation criteria. Gynecol Endocrinol. 2007;23(3):153-60. 10.1080/09513590701214158 17454169

[15] . Soares EM, Azevedo GD, Gadelha RG, Lemos TM, Maranhão TM. Prevalence of the metabolic syndrome and its components in Brazilian women with polycystic ovary syndrome. Fertil Steril. 2008;89(3):649-55. 10.1016/j.fertnstert.2007.03.081 17543961

[16] . Ni RM, Mo Y, Chen X, Zhong J, Liu W, Yang D. Low prevalence of the metabolic syndrome but high occurrence of various metabolic disorders in Chinese women with polycystic ovary syndrome. Eur J Endocrinol. 2009;161(3):411-8. 10.1530/EJE-09-0298 19542239

[17] . Park HR, Choi Y, Lee HJ, Oh JY, Hong YS, Sung YA. The metabolic syndrome in young Korean women with polycystic ovary syndrome. Diabetes Res Clin Pract. 2007;77 Suppl 1:S243-6. 10.1016/j.diabres.2007.01.065 17610984

[18] . Vural B, Caliskan E, Turkoz E, Kilic T, Demirci A. Evaluation of metabolic syndrome frequency and premature carotid atherosclerosis in young women with polycystic ovary syndrome. Hum Reprod. 2005;20(9):2409-13. 10.1093/humrep/dei100 15919774

[19] . Carmina E, Napoli N, Longo RA, Rini GB, Lobo RA. Metabolic syndrome in polycystic ovary syndrome (PCOS): lower prevalence in southern Italy than in the USA and the influence of criteria for the diagnosis of PCOS. Eur J Endocrinol. 2006;154(1):141-5. 10.1530/eje.1.02058 16382003

[20] . Rotterdam ESHRE/ASRM-Sponsored PCOS consensus workshop group. Revised 2003 consensus on diagnostic criteria and long-term health risks related to polycystic ovary syndrome (PCOS). Hum Reprod. 2004;19(1):41-7. 10.1093/humrep/deh098 14688154

[21] . National Cholesterol Education Program (NCEP) Expert Panel on Detection, Evaluation, and Treatment of High Blood Cholesterol in Adults (Adult Treatment Panel III). Third Report of the National Cholesterol Education Program (NCEP) Expert Panel on Detection, Evaluation, and Treatment of High Blood Cholesterol in Adults (Adult Treatment Panel III) final report. Circulation. 2002;106(25):3143-421. 12485966

[22] . Ferriman D, Gallwey JD. Clinical assessment of body hair growth in women. J Clin Endocrinol Metab. 1961;21:1440-7. 10.1210/jcem-21-11-1440 13892577

[23] . Standards of medical care in diabetes – 2012. Diabetes Care. 2012;35 Suppl 1:S11-63. 10.2337/dc12-s011 PMC3632172 22187469

[24] . Matthews DR, Hosker JP, Rudenski AS, Naylor BA, Treacher DF, Turner RC. Homeostasis model assessment: insulin resistance and beta-cell function from fasting plasma glucose and insulin concentrations in man. Diabetologia. 1985;28(7):412-9. 10.1007/BF00280883 3899825

[25] . Ehrmann DA, Liljenquist DR, Kasza K, Azziz R, Legro RS, Ghazzi MN; PCOS/Troglitazone Study Group. Prevalence and predictors of the metabolic syndrome in women with polycystic ovary syndrome. J Clin Endocrinol Metab. 2006;91(1):48-53. 10.1210/jc.2005-1329 16249284

[26] . Vrbíková J, Vondra K, Cibula D, Dvoráková K, Stanická S, Srámková D, et al. Metabolic syndrome in young Czech women with polycystic ovary syndrome. Hum Reprod. 2005;20(12):3328-32. 10.1093/humrep/dei221 16085666

[27] . Miccoli R, Bianchi C, Odoguardi L, Penno G, Caricato F, Giovannitti MG, et al. Prevalence of the metabolic syndrome among Italian adults according to ATP III definition. Nutr Metab Cardiovasc Dis. 2005;15(4):250-4. 10.1016/j.numecd.2004.09.002 16054548

[28] . Hildrum B, Mykletun A, Hole T, Midthjell K, Dahl AA. Age-specific prevalence of the metabolic syndrome defined by the International Diabetes Federation and the National Cholesterol Education Program: the Norwegian HUNT 2 study. BMC Public Health. 2007;7:220. 10.1186/1471-2458-7-220 PMC2048947 17727697

[29] . Park YW, Zhu S, Palaniappan L, Heshka S, Carnethon MR, Heymsfield SB. The metabolic syndrome: prevalence and associated risk factor findings in the US population from the Third National Health and Nutrition Examination Survey, 1988-1994. Arch Intern Med. 2003;163(4):427-36. 10.1001/archinte.163.4.427 PMC3146257 12588201

[30] . Boden G, Chen X, DeSantis RA, Kendrick Z. Effects of age and body fat on insulin resistance in healthy men. Diabetes Care. 1993;16(5):728-33. 10.2337/diacare.16.5.728 8495612

[31] . Marcondes JA, Hayashida SA, Barcellos CR, Rocha MP, Maciel GA, Baracat EC. Metabolic syndrome in women with polycystic ovary syndrome: prevalence, characteristics and predictors. Arq Bras Endocrinol Metabol. 2007;51(6):972-9. 10.1590/s0004-27302007000600012 17934665

[32] . Espinós-Gómez JJ, Rodriguez-Espinosa J, Ordóñez-Llanos J, Calaf-Alsina J. Metabolic syndrome in Mediterranean women with polycystic ovary syndrome: when and how to predict its onset. Gynecol Endocrinol. 2012;28(4):264-8. 10.3109/09513590.2011.613968 21962027

